# Hepatitis B virus reactivation in patients with hematologic malignancies treated with Bruton tyrosine kinase inhibitors

**DOI:** 10.1007/s44313-025-00093-3

**Published:** 2025-08-15

**Authors:** Joon Young Hur, Jung-Hee Lee, Je-Hwan Lee, Han-Seung Park, Hyunkyung Park, Yunsuk Choi, Jung Hye Choi, Young-Woong Won, Sang Eun Yoon, Won Seog Kim, Seok Jin Kim

**Affiliations:** 1 Department of Hematology, Asan Medical Center, University of Ulsan College of Medicine, Seoul, Korea; 2 Department of Internal Medicine, Hanyang University College of Medicine, Hanyang University Guri Hospital, Guri, Korea; 3 Division of Hematology-Oncology, Department of Medicine, Samsung Medical Center, Sungkyunkwan University School of Medicine, 81, Irwon-ro Gangnam-gu, Seoul, 06351 Korea

**Keywords:** Hepatitis B, Ibrutinib, Zanubrutinib, Chronic lymphocytic leukemia, Mantle cell lymphoma

## Abstract

**Purpose:**

Bruton tyrosine kinase inhibitors (BTKis) are effective and well-tolerated treatments for chronic lymphocytic leukemia (CLL) and mantle cell lymphoma (MCL). Here, we describe the clinical characteristics of hepatitis B virus (HBV) reactivation in patients with hematological malignancies treated with BTKis.

**Methods:**

Patients were required to have a pathologically confirmed diagnosis of CLL or MCL, receive at least one cycle of ibrutinib or zanubrutinib, and have either positive hepatitis B surface antigen or hepatitis B core antibody at diagnosis. Patients were excluded if they had received rituximab or obinutuzumab within the previous 12 months.

**Results:**

We identified five patients with CLL and one with MCL who had resolved HBV infections and received BTKis during the study period. None of the patients received anti-HBV prophylaxis after CLL diagnosis. The patient with MCL who received zanubrutinib was confirmed to have HBV reactivation even after prophylactic entecavir administration followed by tenofovir. All five patients with CLL received ibrutinib as second-line therapy. A 62-year-old man died of hepatorenal syndrome associated with HBV reactivation despite entecavir treatment.

**Conclusion:**

To the best of our knowledge, this is the first description of HBV-related death in patients receiving BTKis from HBV-endemic areas, and the first case of HBV reactivation associated with zanubrutinib despite previous entecavir prophylaxis. Further prospective studies are warranted to develop useful guidelines for monitoring HBV DNA and antiviral prophylaxis to prevent HBV reactivation after BTKi therapy.

## Background

In Korea, the number of patients with chronic hepatitis B has consistently increased over the past 15 years [1]. The risk of hepatitis B virus (HBV) reactivation during rituximab treatment in patients with B-cell lymphoma is a well-known serious complication in patients with seropositive hepatitis B surface antigen (HBsAg) and those with resolved HBV infection (seronegative HBsAg and seropositive hepatitis B core antibody [HBcAb]). Antiviral prophylaxis is strongly recommended for patients with seropositive HBsAg or HBcAb who receive rituximab.

Selective Bruton tyrosine kinase (BTK) inhibitors, such as ibrutinib and zanubrutinib, have been widely used as effective and well-tolerated treatments for chronic lymphocytic leukemia (CLL) [2] and mantle cell lymphoma (MCL) [3]. Moreover, they are used to treat Waldenström macroglobulinemia [4], marginal zone lymphoma [5], and chronic graft-versus-host disease after the failure of one or more lines of systemic therapy [6]. By blocking BTK, which is crucial for B cell development and acts on multiple anti-apoptotic signaling pathways, ibrutinib has been under development and has shown remarkable efficacy in several clinical trials [7]. Previous case reports have described HBV reactivation in patients with CLL who received ibrutinib [8–11]. All of these patients recovered after discontinuation of ibrutinib and showed favorable progress. However, only a few reports on HBV reactivation in patients with MCL receiving zanubrutinib have been published in the literature. Thus, in this multicenter study in Korea, where HBV is prevalent, we describe the fatal side effects of hepatic failure associated with HBV reactivation in patients with hematologic malignancies treated with BTK inhibitors.

## Materials and methods

### Patients

The study cohort consisted of patients diagnosed with CLL or MCL at Hanyang University Guri Hospital, Samsung Medical Center, and Asan Medical Center between January 2010 and August 2024. The inclusion criteria for this study included patients with confirmed pathological diagnosis of CLL or MCL, and patients who had received at least one cycle of BTK inhibitors and had either positive HBsAg or HBcAb at diagnosis. Patients who received BTK inhibitors were further classified and those with hepatitis B reactivation were enrolled. Patients were excluded if they had received rituximab or obinutuzumab within the previous 12 months or if they had no data on demographics, HBV characteristics, stage (CLL, MCL), previous therapy (CLL, MCL), and HBV-associated clinical outcomes (fulminant hepatitis, liver failure, liver cirrhosis, or death).

### Definition

Resolved HBV infection was defined as the absence of HBsAg, presence of HBcAb, and/or undetectable HBV DNA levels [12]. In patients with resolved HBV infection, HBV reactivation was characterized by an increase in the alanine transaminase (ALT) level to ≥ 3 times the baseline level and > 100 U/L. HBV reactivation was also defined as an increase in HBV DNA (> 1,000 IU/mL) in patients who had previously presented with positive HBV DNA, the reappearance of HBV DNA in a patient who previously had no HBV DNA, and as the reappearance of HBsAg-positive conversion. HBV-associated fulminant hepatitis or acute liver failure was defined by an international normalized ratio (INR) > 1.5 and hepatic encephalopathy following HBV reactivation [13, 14]. Critical illness is a severe medical condition that threatens life and often requires intensive medical or mechanical support for vital organ function.

The follow-up period was defined as the time from the initiation of BTK inhibitor therapy until March 31, 2025 (the end of data collection), even for patients who no longer receive BTK inhibitors.

## Results

### Patient characteristics

We identified five patients with CLL who had resolved HBV infection and received ibrutinib during the study period. All five patients with CLL received ibrutinib as a second-line therapy. Among the five patients with CLL, three received anti-CD20 monoclonal antibody (rituximab or obinutuzumab) before ibrutinib. None of the five patients received anti-HBV prophylaxis after the diagnosis of CLL. Additionally, we identified one patient with MCL who had a resolved HBV infection and received zanubrutinib. The patient with MCL was confirmed to have HBV reactivation even after prophylactic entecavir administration followed by tenofovir.

The demographics and characteristics of all patients with positive anti-HBcAb and negative HBsAg before ibrutinib treatment are shown in Table 1.

**Table 1:** Characteristics of the patients who developed HBV reactivation

	Age (sex)	Time from initiation of BTKi to HBV reactivation (months)	Baseline hepatic marker	HBsAg sero-conversion	Bilirubin (mg/dL)	HBV DNA (IU/mL)	Clinical outcome
1	81 (F)	12	HBsAg (−), HBsAb (+), and HBcAb (+)	Yes	3	> 9 × 10^8^	Undetectable HBV DNA level after 6 months of tenofovir
2	62 (M)	22	HBsAg (−), HBsAb (+), and HBcAb (+)	Yes	3.5	445,000,000	Death
3	63 (M)	7	HBsAg (−), HBsAb (+), and HBcAb (+)	Yes	15.6	52,600,000	Critically ill
4	60 (F)	20	HBsAg (−), HBsAb (+), and HBcAb unknown	Yes	31.4	420,000,000	Critically ill
5	57 (M)	6	HBsAg (−), HBsAb (−), and HBcAb (+)	No	2.6	13,200,000	Suboptimal HBV control after 6 months
6	63 (M)	8	HBsAg (−), HBsAb (+), and HBcAb (+)	Yes	1.4	38,000,000	Suboptimal HBV control after 2 months

### First case

**Fig. 1:**
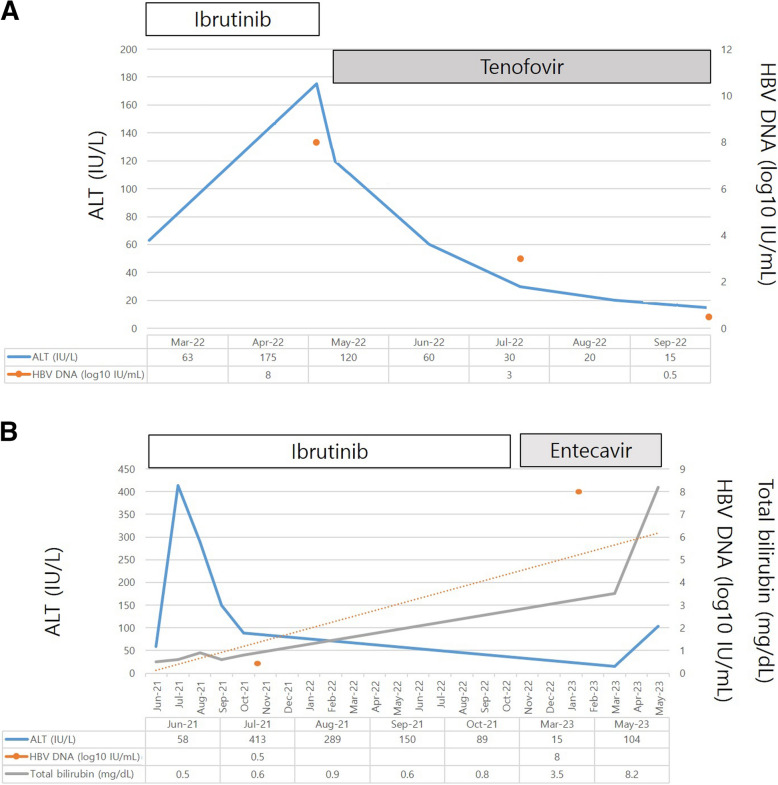
HBV reactivation in patients who received ibrutinib. **A**. Patients who recovered from HBV reactivation following tenofovir treatment. **B**. Patients who did not recover after HBV reactivation, despite treatment with entecavir

An 81-year-old woman was diagnosed with CLL (Rai stage III, *TP53* FISH [-]). After 16 months of chlorambucil treatment, CLL progressed, and ibrutinib therapy was initiated. At the time of ibrutinib initiation, the HBV serum markers were negative for HBsAg and positive for hepatitis B surface antibody (HBsAb) and HBcAb. The patient was intermittently treated with ibrutinib because of recurrent infections and diarrhea. Twelve months after ibrutinib therapy, the patient developed HBV reactivation with increased alanine transaminase levels (Fig. 1). HBV serology revealed HBsAg seropositivity, hepatitis B e-antigen (HBeAg) seropositivity, and HBV DNA levels of > 9 × 10^8^ IU/mL. Ibrutinib was discontinued, and tenofovir 25 mg was initiated (Fig. 1A). After 3 months of tenofovir treatment, the HBV DNA level decreased to 5,418 IU/mL, and ibrutinib was restarted. Despite undetectable HBV DNA levels after 6 months of tenofovir treatment, the patient died of COVID-19.

### Second case

A 62-year-old man was diagnosed with CLL (Rai stage I, *t(11;14)* FISH [-]) in February 2017. At the time of diagnosis, the HBV serum markers were negative for HBsAg and positive for HBsAb and HBcAb. After observation without treatment, the number of cervical lymph nodes increased, and chemotherapy (obinutuzumab + chlorambucil) was initiated in March 2018. In cycle one, obinutuzumab 100 mg was administered on day 1, followed by obinutuzumab 1,000 mg on day 8, and chlorambucil 0.5 mg/kg on day 15. Chemotherapy (obinutuzumab + chlorambucil), administered every 4 weeks, was terminated after six cycles, achieving complete remission. In March 2021, lymph node progression was noted, and the patient was intermittently treated with ibrutinib. After 22 months of ibrutinib therapy, the patient developed ascites and liver cirrhosis. Consequently, HBV reactivation was observed, with an increase in liver enzyme levels. Positive conversion of HBsAg with an increase in ALT levels and the appearance of HBV DNA were observed (serum HBV DNA level: 445,000,000 IU/mL; Fig. 1B). Four months after being diagnosed with decompensated liver cirrhosis associated with HBV reactivation, the patient died of hepatorenal syndrome despite entecavir treatment.

### Third case

A 63-year-old man was diagnosed with CLL (Rai stage I, Binet stage A) in March 2018. At the time of diagnosis, the HBV serum markers were negative for HBsAg and positive for HBsAb and HBcAb. Four years after diagnosis without treatment, multiple lymph nodes were enlarged, and the lymphocyte count increased to 17,656/mm^3^. Chemotherapy (obinutuzumab + chlorambucil) was initiated in March 2022. The chemotherapy was terminated after six cycles, and complete remission was achieved. In January 2024, progression of the lymph nodes (retroperitoneal, small bowel mesentery, iliac chains, and inguinal) was noted, and the patient was treated with ibrutinib 420 mg. After seven months of ibrutinib therapy, HBsAg was positively converted with the appearance of HBV DNA (serum HBV DNA level: 52,600,000 IU/mL), and an increased total bilirubin level of 15.6 mg/dL (direct bilirubin: 13.3 mg/dL) was noted. An ALT level of 276 U/L, PT/INR value of 1.88, albumin level of 3.0 g/dL, and ammonia level of 95.7 µmol/L were noted. After being diagnosed with fulminant hepatitis B associated with HBV reactivation, the patient became critically ill despite receiving tenofovir treatment.

### Fourth case

A 60-year-old woman was diagnosed with CLL (Rai stage I, Binet stage A) in 2011. After 12 years of chlorambucil treatment, CLL progressed, and ibrutinib was initiated in 2023. At the time of ibrutinib initiation, the HBV serum markers were negative for HBsAg and positive for HBsAb. After 20 months of ibrutinib therapy, HBsAg was positively converted with the appearance of HBV DNA (serum HBV DNA level: 420,000,000 IU/mL), and an increased total bilirubin level of 31.4 mg/dL (direct bilirubin: 29.7 mg/dL) was noted. Further, an ALT level of 548 U/L, a PT/INR value of 1.32, and an albumin level of 2.8 g/dL were noted. After being diagnosed with fulminant hepatic failure associated with HBV reactivation, the patient became critically ill despite receiving tenofovir treatment.

### Fifth case

A 57-year-old man was diagnosed with CLL (Rai stage IV, Binet stage C) in May 2020. Flow cytometry (chronic leukemia immunophenotyping) revealed CD5 positivity, CD23 positivity, Sm Ig weak positivity, CD22 or CD79b weak, CD10 negativity, and FMC7 negativity. At the time of diagnosis, the HBV serum markers were negative for HBsAg and HBsAb and positive for HBcAb. After undergoing a rituximab + fludarabine + cyclophosphamide (RFC) treatment regimen for 24 months, the disease progressed, and ibrutinib was initiated. At the time of ibrutinib initiation, the HBV serum markers were negative for HBsAg and HBsAb and positive for HBcAb. Six months after ibrutinib therapy, the patient developed HBV reactivation (serum HBV DNA level: 13,200,000 IU/mL) without HBsAg-positive conversion. An immune escape variant was suggested. The patient has been treated with rituximab and venetoclax since February 2024, after changing the drug to chlorambucil.

### Sixth case

A 63-year-old man was diagnosed with MCL with a blastoid variant (stage III) in February 2022. At the time of diagnosis, the HBV serum markers were negative for HBsAg and HBsAb and positive for HBcAb. After four cycles of the R-hyperCVAD (rituximab + cyclophosphamide + doxorubicin + vincristine + dexamethasone/rituximab + methotrexate + cytarabine) regimen, the patient underwent autologous stem cell transplantation (ASCT) with busulfan, etoposide, and cyclophosphamide (BuCyE) in March 2023 with entecavir prophylaxis. At the time of ASCT, the HBV serum markers were negative for HBsAg and HBsAb. After ASCT treatment for 13 months, the disease progressed, and zanubrutinib was initiated. At the time of zanubrutinib initiation, the HBV serum markers were negative for HBsAg and HBsAb. HBV reactivation prophylaxis with entecavir was continued. After 8 months of zanubrutinib therapy, the patient developed HBV reactivation (serum HBV DNA level: 38,000,000 IU/mL). Associated findings included HBsAg-positive conversion, an increased total bilirubin level of 1.4 mg/dL, an albumin level of 3.4 g/dL, and an ALT level of 1481 U/L. After 3 months of tenofovir treatment, HBV DNA decreased to 12 IU/mL, and zanubrutinib was restarted.

## Discussion

To the best of our knowledge, this is the first study to show that patients with hematological malignancies receiving BTK inhibitors may develop fulminant hepatitis, which may result in death due to HBV reactivation. Before administering ibrutinib, it is necessary to check for the presence of HBV infection. Prophylaxis for HBV reactivation during treatment with ibrutinib or zanubrutinib has not yet been established. In a previous study, no Chinese patients with resolved HBV infection showed HBV reactivation after ibrutinib treatment [15]. Furthermore, ibrutinib could be safely administered after antiviral control of HBV reactivation [16]. Long-term RESONATE-2 data, including those of most Western patients, showed sustained survival benefits and no new safety signals [2, 17]. However, regular monitoring of HBV reactivation markers and prophylaxis of HBV reactivation are required because Korean patients with HBV reactivation had a fatal course. In addition, given that ALT levels were not significantly elevated in patients receiving ibrutinib, screening for HBV reactivation in HBsAg-negative/HBcAb-positive patients may be sufficiently achieved with HBsAg or HBV DNA follow-up during routine hospital visits.

HBV infection rates were the highest in the Western Pacific region (Taiwan, Japan, South Korea, and China), with 6.2% of the adult population being infected [18]. Given the higher rate of HBV carriers among Korean patients, the variable results of previous studies might be associated with their ethnic nature. In addition to the already known side effects of ibrutinib, such as diarrhea, hypertension, bleeding (major hemorrhage), opportunistic infections [19], and atrial fibrillation [2], it is worth paying attention to the side effects of HBV reactivation.

HBV reactivation during BTK inhibitor treatment is presumed to be possible because it inhibits the B-cell receptor signaling pathway, leading to strong immunosuppression. Further prospective studies confirming that drugs, such as entecavir or lamivudine, can effectively prevent ibrutinib-induced HBV reactivation are urgently needed. In the era of ibrutinib as the first-line treatment for patients with CLL, HBcAb positivity is considered a potential risk factor for HBV reactivation in patients with HBsAg-negativity.

In conclusion, this is the first description of HBV-related deaths in patients receiving BTK inhibitors in HBV-endemic areas, and the first description of HBV reactivation associated with zanubrutinib, despite previous entecavir prophylaxis. Further prospective studies are warranted to develop useful guidelines for HBV DNA monitoring and antiviral prophylaxis to prevent HBV reactivation after treatment with BTK inhibitors.

## Data Availability

Data are available upon reasonable request.
